# Indirect exposure to socially defeated conspecifics using recorded video activates the HPA axis and reduces reward sensitivity in mice

**DOI:** 10.1038/s41598-020-73988-z

**Published:** 2020-10-09

**Authors:** Yuko Nakatake, Hiroki Furuie, Masatoshi Ukezono, Misa Yamada, Kazumi Yoshizawa, Mitsuhiko Yamada

**Affiliations:** 1 Department of Neuropsychopharmacology, National Institute of Mental Health, National Center of Neurology and Psychiatry, 4-1-1 Ogawahigashimachi, Kodaira, Tokyo 187-8553 Japan; 2 Laboratory of Pharmacology and Therapeutics, Faculty of Pharmaceutical Sciences, Tokyo University of Science, 2641, Yamazaki, Noda, Chiba 278-8510 Japan; 3 Developmental Disorder Data Multi-Level Integration Unit Medical Science Innovation Hub Program, RIKEN, 4-1-1 Kizugawadai, Kizugawa, Kyoto 619-0225 Japan

**Keywords:** Animal behaviour, Trauma

## Abstract

Rodents perceive the emotional states of conspecifics using vision. In the present study, we demonstrated that exposure to the video-recorded distress of conspecifics induces stress responses in male C57BL/6J mice. A single exposure to a video-recorded scene of the social defeat stress (SDS) increased plasma corticosterone levels in these mice. This physiological change was suppressed by blocking the visual information, suggesting that vision plays a crucial role in inducing stress responses. Furthermore, after exposure to the video, there were increased numbers of c-Fos-positive neurons in the anterior cingulate cortex and other brain areas that are associated with the negative valence and empathy systems, but not in the regions related to the pain signaling. In addition, repeated exposure to SDS videos induced an apparent reduction in reward sensitivity in the sucrose preference test, but did not affect avoidance behaviour in the social interaction test or immobility behaviour in the forced swim test. Reduced reward sensitivity in mice reflects anhedonia, which is a core symptom of depression in humans. Our video SDS model therefore provides a unique opportunity to not only understand the mechanisms underlying stress-induced anhedonia, but also to screen effective candidate molecules for stress-related disorders with greater reproducibility.

## Introduction

Exposure to traumatic events, regardless if it is direct or indirect, causes severe mental damage to individuals and may be a risk factor for developing post-traumatic stress disorder^1,2^. Visually broadcast news reports of disasters (e.g., earthquakes and tsunamis) or unpleasant situations (e.g., terrorism) may also hurt many people simultaneously. A recent study of the Boston Marathon bombings demonstrated that indirect exposure through the media resulted in higher acute stress symptom scores than direct exposure^3^, suggesting that indirectly experiencing stressful stimuli has more of an impact on mental and physical health than was originally thought.

Recent studies have successfully established animal models of vicarious stress, such as a witness foot shock model and a witness social defeat stress (SDS) model, by adding the concept of witnessing^4,5^. In these studies, animals witnessing the stressed conspecifics display stress-related behavioural and physiological changes that are similar to those of actually stressed animals^5–10^. We have also recently reported that the emotional stress caused by witnessing SDS induces an apparent reduction in reward sensitivity, while experiencing SDS has no such effect^10^.

Rodents perceive the emotional reactions of conspecifics using vision, and their behaviours are influenced by the emotional state of the conspecific. For example, rats^11^ and mice^12–14^ display fear response such as freezing behaviours when they observe their conspecifics exposed to foot shock. In addition, rats that observe their cage-mates in pain display altered pain sensitivity^15^. It has also been reported that rats are able to discriminate between images of other rats’ painful and neutral facial expressions, and tend to avoid the former^16^. These findings suggest that the presentation of recorded negative visual images might stimulate negative emotions in rodents. However, it remains unclear whether rodents feel stressed when visually presented with recordings of conspecifics in fear and distress.

In the present study, we therefore investigated whether exposure to the recorded distress of conspecifics is a stressful stimulus that induces physiological and behavioural changes in male C57BL/6J mice. First, we recorded SDS scenes and examined the effects of a single SDS video exposure on peripheral corticosterone levels to detect stress responses in mice. Then, we examined c-Fos expression in the brain as a marker for neuronal activity. Finally, we investigated the effects of 10 days of SDS video exposure on emotional behaviour using the social interaction test (SIT), elevated plus-maze test (EPM), forced swim test (FST), and sucrose preference test (SPT).

## Results

### Experiment 1: Single exposure to SDS video

#### Exposure to SDS video increases plasma corticosterone levels in mice

**Figure 1:**
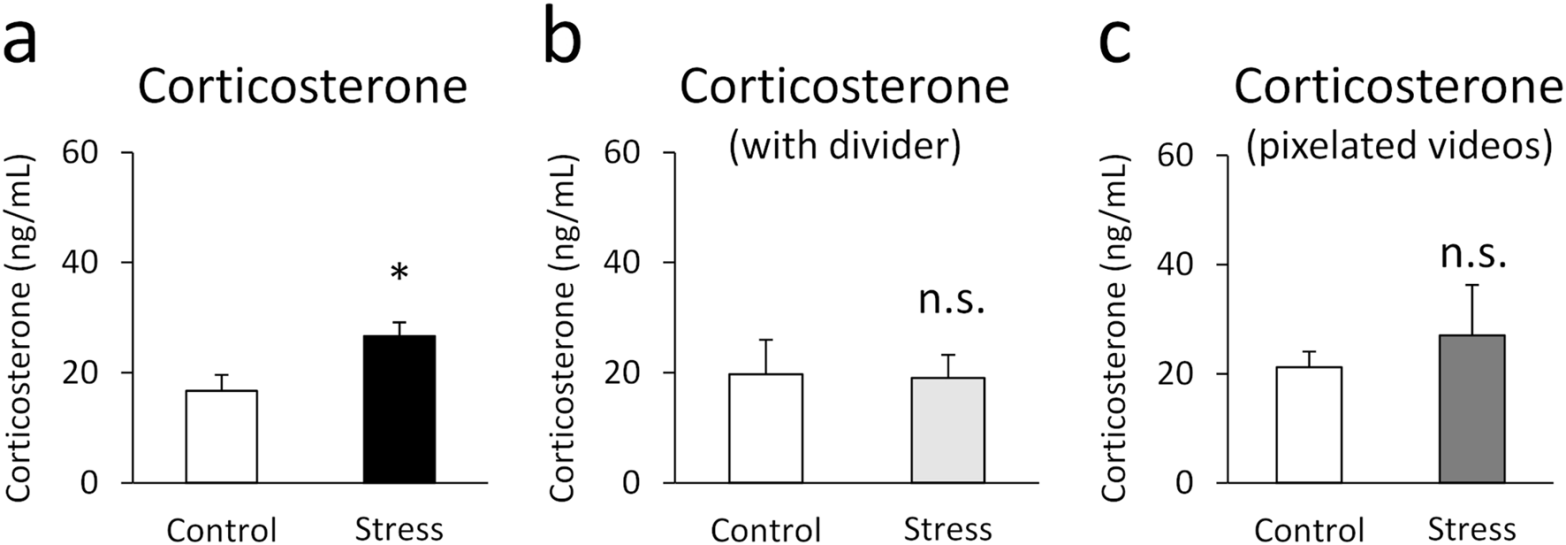
A single exposure to the SDS video increased plasma levels of corticosterone. (**a**) The levels of corticosterone 20 min after a single video exposure. (**b**) The levels of corticosterone when visual information was blocked by the opaque divider during the video exposure. (**c**) The levels of corticosterone when mice were exposed to the pixelated SDS video. All data are presented as mean ± SEM. *p < 0.05 (n = 5 per group).

We first investigated whether the SDS video was a stressor that increased corticosterone levels in mice. Figure 1 shows the plasma levels of corticosterone 20 min after a single video exposure. A Student’s *t*-test revealed a significant difference between the control and stress groups (*t*(8) = 2.343, *p* = 0.047). Mice exposed to the SDS video had significantly increased plasma corticosterone levels compared with controls (Fig. 1a). No obvious behavioural changes were observed.

However, when visual information was blocked by the opaque divider and only sound cues were available, there were no significant differences between the control and stress groups (*U* = 8.000, *p* = 0.347) (Fig. 1b). In addition, when the content of SDS and control video was masked by pixelating, there were no significant differences in the corticosterone levels between the groups (*U* = 10.000, *p* = 0.602) (Fig. 1c).

#### Exposure to SDS video increases c-Fos-positive neurons in a range of brain areas

**Figure 2:**
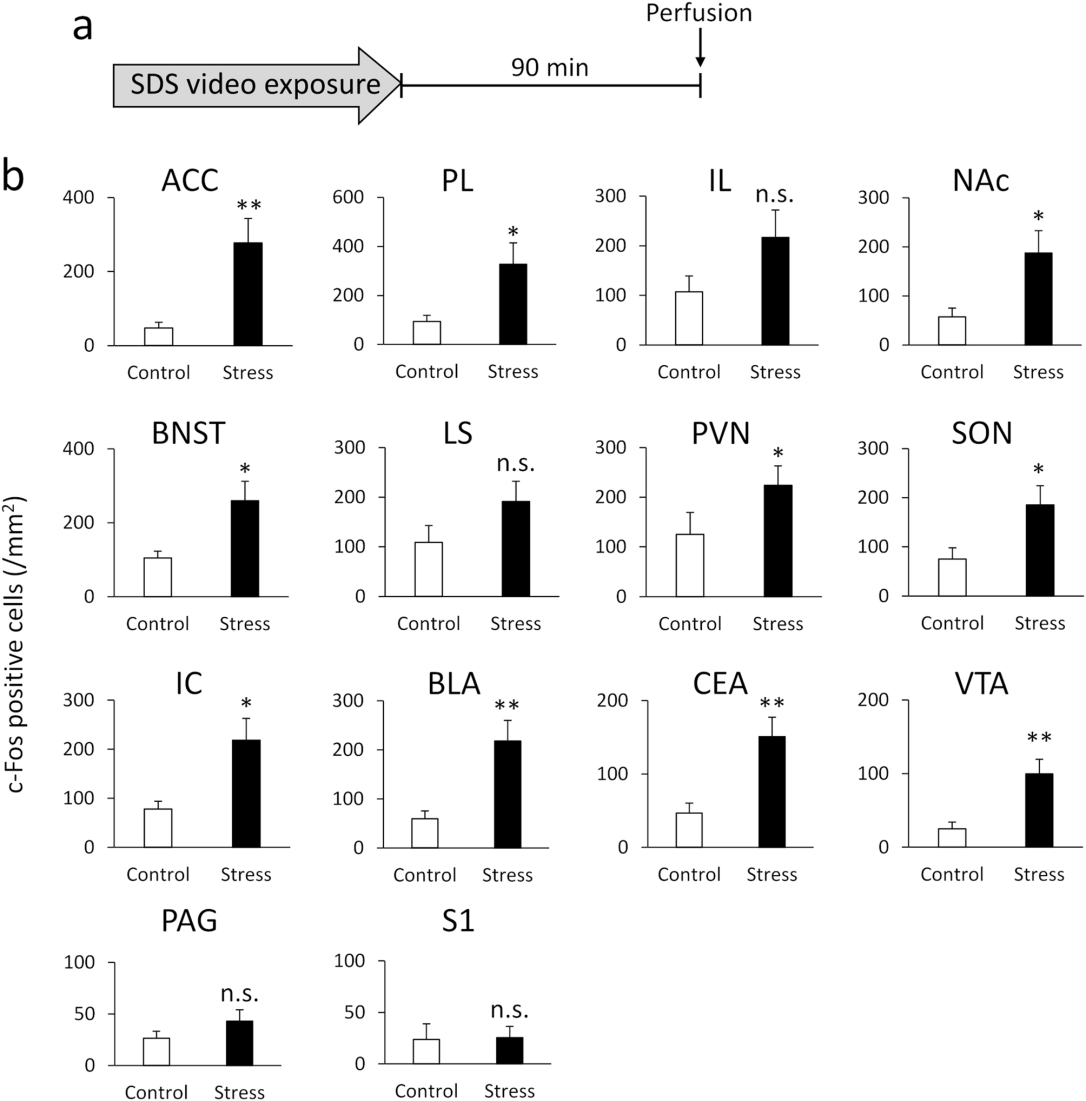
A single exposure to the SDS video increased c-Fos expression in a range of brain areas. (**a**) The schedule of video exposure and the subsequent experiments. Mice were sacrificed for immunohistochemistry 90 min after exposure to the SDS video. (**b**) The numbers of c-Fos-positive neurons in the anterior cingulate cortex (ACC), prelimbic cortex (PL), infralimbic cortex (IL), nucleus accumbens (NAc), bed nucleus of the stria terminalis (BNST), lateral septum (LS), hypothalamic paraventricular nucleus (PVN), supraoptic nucleus (SON), insular cortex (IC), basolateral amygdala (BLA), central amygdala (CeA), ventral tegmental area (VTA), periaqueductal gray (PAG), and primary somatosensory cortex (S1). All data are presented as mean ± SEM. *p < 0.05, **p < 0.01 (control group: n = 10; stress group: n = 12).

We next investigated which brain areas were activated by a single SDS video exposure. Figure 2b shows the numbers of c-Fos-positive neurons 90 min after a single SDS video exposure. The Mann–Whitney *U* test and unpaired *t*-test revealed significant differences between the stress and control groups in the anterior cingulate cortex (ACC; *t*(12.146) = 3.223, *p* = 0.007), prelimbic cortex (PL; *t*(12.863) = 2.471, *p* = 0.028), nucleus accumbens (NAc; *t*(20) = 2.410, *p* = 0.026), bed nucleus of the stria terminalis (BNST; *t*(13.517) = 2.660, *p* = 0.019), hypothalamic paraventricular nucleus (PVN; *U* = 29.500, *p* = 0.044), supraoptic nucleus (SON; *t*(20) = 2.209, *p* = 0.039), insular cortex (IC; *t*(13.801) = 2.864, *p* = 0.013), basolateral amygdala (BLA; *t*(14.096) = 3.355, *p* = 0.005), central amygdala (CeA; *t*(16.082) = 3.349, *p* = 0.004), and ventral tegmental area (VTA; *t*(15.293) = 3.293, *p* = 0.005). There were no significant differences in the infralimbic cortex (IL; *t*(20) = 1.557, *p* = 0.135), lateral septum (LS; *t*(20) = 1.451, *p* = 0.162), periaqueductal gray (PAG; *t*(20) = 1.148, *p* = 0.264), and primary somatosensory cortex (S1; *U* = 51.000, *p* = 0.473). Thus, SDS video exposure induced significantly increased neuronal activation in the ACC, PL, NAc, BNST, PVN, SON, IC, BLA, CeA, and VTA compared with the control group.

### Experiment 2: Repeated exposure to SDS videos

#### Repeated exposure to SDS videos reduces weight gain and sucrose preference

**Figure 3:**
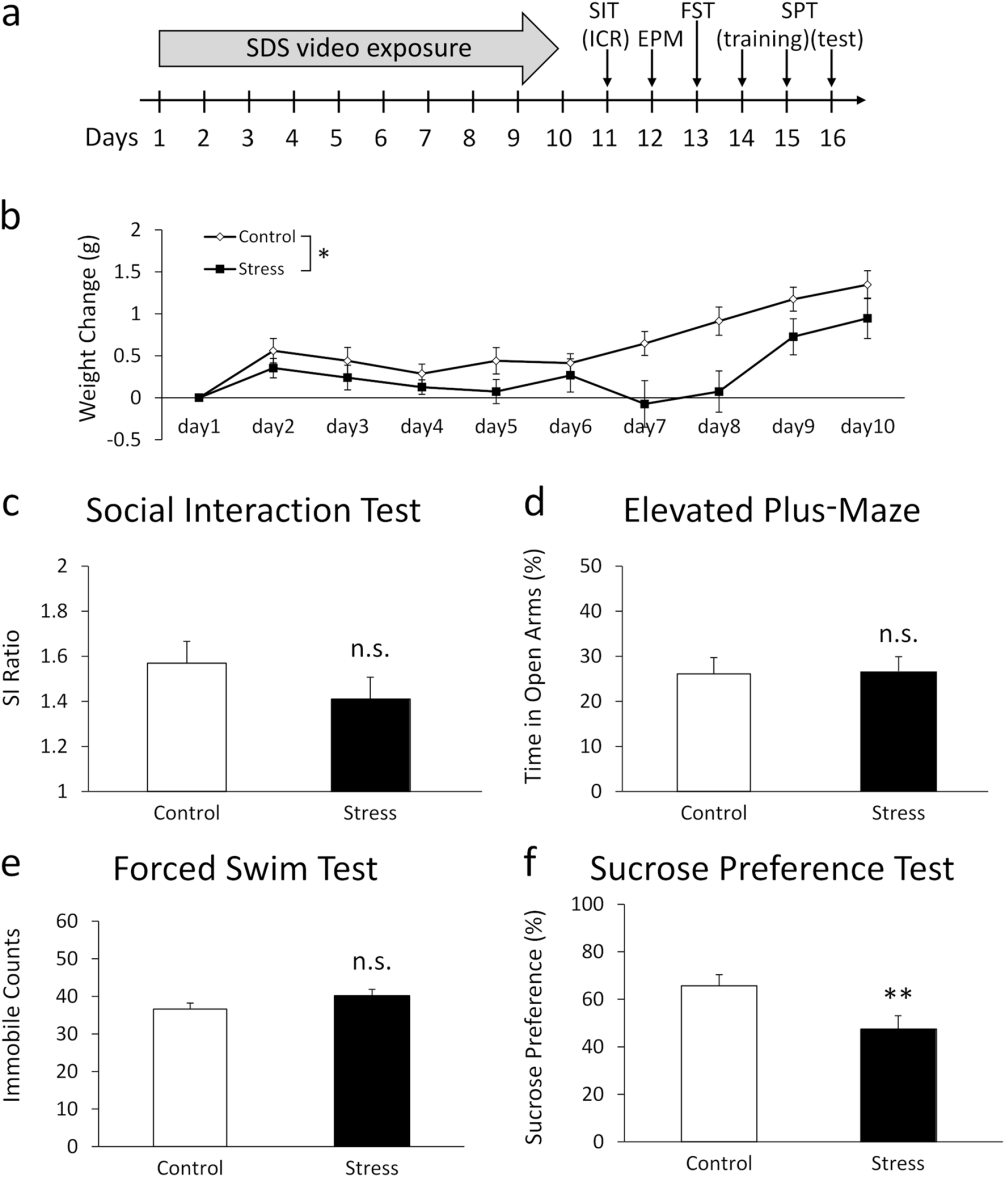
Repeated exposure to the SDS videos for 10 days decreased weight gain and sucrose preference. (**a**) The schedule of video exposure and the subsequent behavioural experiments. (**b**) The changes in body weight gain during the video exposure period. (**c**) The changes in the social interaction ratio in the social interaction test. (**d**) The percentage of time spent in the open arms in the elevated-plus maze test. (**e**) The immobile counts in the forced swim test. (f) The sucrose preference percentage in the sucrose preference test. All data are presented as mean ± SEM. *p < 0.05, **p < 0.01 (n = 15 per group; except for the forced swim test, where a failure in recording led to control group: n = 13 and stress group: n = 15).

To further investigate the effects of SDS video exposure, we repeatedly exposed the mice to SDS videos and assessed their daily body weight and emotional behaviours. Figure 3b shows the weight gain during the stress exposure period of 10 days. Changes in body weight were calculated by subtracting the weight on day 1 of SDS video exposure from the daily body weight. There were no significant differences in the initial body weight between the groups (control: 23.93 ± 0.40; stress: 23.51 ± 0.50; *t*(28) = 0.600, *p* = 0.553). Two-way ANOVA revealed a significant main effect of stress (*F*(1, 28) = 4.926, *p* = 0.035) and day (*F*(4.45, 124.59) = 13.273, *p* < 0.001), but there was no significant interaction effect between stress × day (*F*(4.45, 124.59) = 1.929, *p* = 0.102). Repeated exposure to SDS videos led to significantly reduced weight gain in mice compared with the control group. In addition, there were no significant differences in the body weight on the day before the training session of the SPT between the groups (control: 25.81 ± 0.50; stress: 24.82 ± 0.50; *t*(28) = 1.475, *p* = 0.151).

The effects of the SDS videos on emotional behaviours were assessed using the SIT, EPM, FST, and SPT. Figure 3c shows the SI ratio in the SIT at 24 h after the last video exposure. During the 5 min SIT, mice exposed to the SDS videos (the stress group) displayed similar SI ratios to those of the control group (control: 1.57 ± 0.10; stress: 1.41 ± 0.10). There was no significant difference between the groups in the SIT (*U* = 72.000, *p* = 0.093). In addition, there were no significant differences in total distance between the groups, regardless of the presence or absence of the target mice (with target: *t*(28) = 1.736, *p* = 0.094; without target: *t*(28) = 1.584, *p* = 0.124) (Table 1).

**Table 1:** The additional information on the social interaction test, the elevated-plus maze test, and the forced swim test after 10 days of video exposure.

		Control	Stress	*P* value
SIT	Total distance (cm) (without target)	1,325.33 ± 63.69	1,167.34 ± 72.32	n.s.
	Total distance (cm) (with target)	1,120.56 ± 56.84	957.88 ± 70.44	n.s.
EPM	Total distance (cm)	1,453.71 ± 72.56	1,355.50 ± 83.86	n.s.
	Time in closed arms (%)	52.78 ± 3.17	48.94 ± 3.25	n.s.
FST	Swimming counts	23.00 ± 1.49	19.67 ± 1.78	n.s.
	Climbing counts	12.38 ± 1.55	12.13 ± 1.37	n.s.

All data are presented as mean ± SEM. (n = 15 per group; except for the forced swim test, where a failure in recording led to control group: n = 13 and stress group: n = 15).

Figure 3d shows the percentage of time spent in the open arms during the EPM, which was conducted 24 h after the SIT. During the 5 min EPM, mice in the stress group spent a similar amount of time in the open arms as mice in the control group (control: 26.11 ± 3.60; stress: 26.65 ± 3.28). An unpaired *t*-test revealed that there was no significant difference between groups in the EPM (*t*(28) = 0.106, *p* = 0.916). In addition, there were no significant differences in total distance and time in the closed arms between the groups (total distance: *t*(28) = 0.856, *p* = 0.399; time in the closed arms: *t*(28) = 0.816, *p* = 0.422) (Table 1).

Figure 3e shows the immobile counts from the FST, which was conducted 24 h after the EPM. During the 6 min FST, the stress group had slight increases in immobility; however, the scores were similar to those of controls (control: 36.62 ± 1.75; stress: 40.20 ± 1.66). An unpaired *t*-test revealed that there was no significant difference between the groups in the FST (*t*(26) = 1.430, *p* = 0.165). In addition, there were no significant differences in swimming and climbing counts between the groups (swimming: *U* = 62.000, *p* = 0.101; climbing: *t*(26) = 0.117, *p* = 0.907) (Table 1).

Figure 3f shows the percentage of sucrose preference in the SPT after the 2-day training period. During the 1 h SPT, the stress group had lower sucrose preference compared with the control group (control: 65.71 ± 3.76; stress: 47.51 ± 4.51). The Mann–Whitney *U* test revealed a significant difference between the groups in the SPT (*U* = 49.000, *p* = 0.008). In addition, using a two-way ANOVA for water and sucrose consumption over 1 h in the SIT, we revealed that there were significant main effects of stress (*F*(1, 28) = 7.653, *p* = 0.010) and taste (*F*(1, 28) = 10.786, *p* = 0.003), and an interaction effect between stress × taste (*F*(1, 28) = 7.520, *p* = 0.011). *Post-hoc* testing revealed no significant difference in water intake between the control and stress groups (control: 0.48 ± 0.09; stress: 0.37 ± 0.04, *p* = 0.270). However, the stress group had significantly lower sucrose intake compared with the control group (control: 0.85 ± 0.11; stress: 0.40 ± 0.07, *p* = 0.002). These findings indicate that repeated exposure to SDS videos induces significant decreases in sucrose preference in the SPT.

## Discussion

In Experiment 1, we demonstrated that male C57BL/6J mice have increased levels of corticosterone after a single exposure to SDS video. This result suggests that visual exposure to the SDS scene is a stressor that may activate the hypothalamic–pituitary–adrenal (HPA) axis in mice. In addition, single exposure to the SDS video led to activation (as measured by c-Fos expression) of a range of brain areas, including the ACC, PL, NAc, BNST, PVN, SON, IC, BLA, CEA, and VTA. This c-Fos expression pattern was similar to that reported after actual SDS^17,18^. These brain areas are reported to be involved in the negative valence system, which regulates fear, anxiety, aversion, depressive mood, and reward sensitivity^19–22^. Our findings suggest that indirect exposure to SDS via video is a stressor that drives the negative valence system in mice. On the other hand, an increase in c-Fos expression was not observed in the PAG and S1, which are related to the pain signalling. The absence of changes in such brain regions may represent the characteristics of the painless SDS video exposure.

Rodents are reported to display various stress-related changes when directly witnessing SDS^5–10^. It has been suggested that they recognise the emotional state of conspecifics in distress as their own stress, using visual, auditory, and chemosensory stimuli as cues. Interestingly, it has been reported that mice do not display stress-induced avoidance behaviour when visual information is blocked while they witness SDS^5,9^. Similarly, in the present study, stress-induced increases in corticosterone levels were suppressed when we blocked the visual information. Our result suggests that vision plays a crucial role in inducing stress-related various changes when exposed to stressful stimuli, either directly or indirectly.

On the other hand, it is previously reported that an integration of auditory and visual information is important for vicarious fear^12^. Therefore, it is possible that the dissociation between the direction of the image and the sound could activate the HPA. However, we have confirmed that the level of corticosterone was not increased by the exposure to pixelated SDS video, even though the direction of the image and the sound did not match. This finding suggests that the corticosterone response in the SDS video observer was not solely due to this dissociation. In addition, our result also suggests that the content, but not the intensity, of the video is important to activate the HPA axis.

It has been reported that rodents express emotion in their facial expressions, and that they change their behaviours by perceiving the emotional responses of conspecifics^15,23^. It is therefore possible that the mice in the present study observed the facial expressions and defeat postures of their conspecifics in the SDS videos, and recognised them as stressful. We hypothesise that empathy may be a mechanism by which the mice were affected by the SDS videos and displayed stress-related changes. Empathy is one of the emotional processes by which individuals are affected by the state of another’s emotions, at least in humans^24^. Rodents are also known to display empathy-like behaviours^12,14^. For example, when mice observe conspecifics receiving electrical shocks, they exhibit freezing behaviours as if they themselves are receiving the shocks. In addition, witnessing another individual in pain activates the ACC neurons, which are the same neurons that experience self-pain at the single-cell level^25^. Furthermore, inactivation of the ACC results in impaired fear transmission^13^. Therefore, ACC activity is likely involved in the empathy-like behaviour. In the present study, c-Fos-positive neurons in the ACC were increased after exposure to a single SDS video, suggesting that the empathy-related circuit was also driven in this model. We therefore speculate that various brain areas associated with negative emotions and the HPA axis were also activated after exposure to the SDS video.

There is another possibility: that the aggressive ICR which attacked the conspecific was the stressful stimulus that affected the subject mice. However, given the SIT results, where the subject C57BL/6J mice did not show avoidance behaviour toward the ICR mice, it is unlikely that the subject C57BL/6J mice were recognising the ICR mice themselves as a fear target.

We have previously reported that witnessing actual SDS induces avoidance behaviour in the SIT and increases immobility in the FST^10^. In the present study, however, repeated exposure to the SDS video did not affect these parameters. This discrepancy may be because the effects of the SDS video exposure are weaker than the effects of directly witnessing the actual SDS. In this study, we did not conduct a daily investigation of the sensory contact that the subject mice had with the aggressive ICR mice across the partition until the stress exposure the next day, although this is usually performed in a standard SDS paradigm^26^. The lack of sensory contact may therefore also contribute to the discrepancy. In addition, we must note that our SDS video did not contain ultrasonic vocalisation^27,28^ or alarm pheromones^29–31^ which are released by rodents under stressful conditions and could induce physiological and behavioural changes in other conspecifics. Since the integration of multiple sensory inputs is important for vicarious fear, the addition of pheromones and ultrasonic vocalizations to the SDS video could induce changes in the SIT and the FST. It is also interesting to examine c-Fos expression in the brain regions that mediate the pheromones or ultrasonic vocalizations in future studies.

In Experiment 2, mice in the stress group had lower gains in body weight during the SDS video exposure period. Interestingly, the SDS video group did not show any large changes in body weight gain until about day 6. One possible reason is that the effects of daily stress gradually accumulated, and changes in body weight gain appeared a few days after the start of video exposure. On the other hand, recovery of body weight of SDS video group seems to be faster in the last 2 days during the video exposure period. The adaptation to the SDS video may explain this tendency.

In the current study, mice in the SDS video group reduced sucrose preference in the SPT. The lower consumption of sucrose solutions may reflect reduced caloric demand. In the present study, the body weight of the stressed mice returned to the control levels on the day before the training session of the SPT. This result suggests that decrease in sucrose consumption found in the SDS video observer was not secondary to the decreased body weight or caloric demands. In addition, it was previously reported that stressed animals also reduced their intake of calorie-free saccharin solutions^32,33^. Furthermore, mice witnessing actual SDS that showed increase in weight gain were also reported to have reduced sucrose preference^10^. Together, these results strongly suggest that exposure to SDS videos has significant effects on reward sensitivity. The mesolimbic dopamine neurons in the VTA are important for regulating the reward system and stress responses^19,21^. The reduced sucrose preference in the SPT caused by 10 days of video exposure might reflect a dysfunction of VTA dopamine neurons, which were activated after just a single acute video exposure (Fig. 2b). Reduced reward sensitivity in mice reflects anhedonia, which is a core symptom of depression and is difficult to completely improve in humans^34^. Our video SDS model may therefore provide a unique opportunity to understand the mechanisms underlying stress-induced anhedonia.

Recently, the low reproducibility of research in biomedical science has been highlighted as a problem^35^. The probable causes are regarded as variability caused by biological factors, differences in experimental environments, and slight differences in experimental procedures. In the actual SDS model, as well as in the previously reported witnessing SDS models, it is difficult to standardise the intensity of stress exposure for the experimental animals. Such factors might contribute to the discrepancies in results among research groups^36^. Using video exposure, however, we can standardise the intensity of the stress. The video SDS model established in the present study might therefore be useful not only for further investigating the stress-induced behavioural and physiological changes, but also for screening effective candidate molecules for stress-related disorders with greater reproducibility.

In conclusion, we demonstrated that exposure to the video-recorded distress of conspecifics is a stressful stimulus that induces physiological and behavioural changes in mice.

## Materials and methods

### Animals

We used male C57BL/6J mice aged 7–8 weeks and male ICR mice aged over 13 weeks (Japan SLC, Shizuoka, Japan). All mice were housed in standard polyolefin plastic cages at 23 ± 1 °C with a 12 h light–dark cycle with lights on at 8:00, and were allowed free access to food and water. The experiments were conducted in compliance with the Guidelines for the Care and use of Laboratory Animals, and received National Center of Neurology and Psychiatry Animal Care and Use Committee (2017014, 2018027) approval. All behavioural experiments were performed between 13:00 and 17:00.

### SDS video preparation

To prepare the SDS video, we performed SDS experiments according to the standard method for mice SDS models^26^. Prior to the SDS experiment, ICR mice were screened for aggressive behaviours toward C57BL/6J mice according to the screening criteria, which we have previously published^10^. For the SDS experiment, an aggressive ICR mouse was placed into a transparent acrylic cage (10 cm × 23 cm × 12 cm) with their bedding, and a C57BL/6J mouse was then placed into the cage. The SDS session was recorded for 5 min from the side of the cage by a video camera, and different ICR and C57BL/6J mice were used for each session. Two independent evaluators assessed the severity of aggressive behaviours in these videos, based on the numbers of attacks and the facial expressions (narrowed eyes) of the ICR mice, and the defeat posture, freezing behaviour, and squealing of C57BL/6J mice. Of the 38 videos, 20 showing highly aggressive behaviours were selected by agreement between the evaluators. These 20 SDS videos were edited to create the ten 10-min videos used in this study.

As controls, we prepared ten 10-min videos in which a pair of C57BL/6J mice interacted in the same transparent acrylic cage. For the habituation video, a single C57BL/6J mouse was recorded in the cage for 10 min. Mice were presented with this habituation video to become accustomed to the equipment and environment before the effects of video exposure were examined.

In addition, we also generated three pixelated videos (the habituation video, control video and SDS video) to mask the contents, while the intensities of those videos were preserved, by pixelating original videos to 8-pixel based images.

### Experiment 1: Single exposure to SDS video

After the habituation session, a C57BL/6J mouse was exposed to a SDS (stress group) or control (control group) video. We examined the effects of this single video exposure on peripheral corticosterone levels, measured 20 min after video exposure, and c-Fos expression in the brain, measured 90 min after video exposure.

Then, we examined the effect of SDS video exposure on peripheral corticosterone levels under the condition when visual information is blocked by the opaque divider and only sound cues were available. In this experiment, after the habituation session with the divider, a mouse was exposed to SDS or control video with the divider.

Finally, we examined the effect of the pixelated SDS video exposure on peripheral corticosterone levels. In this experiment, after the habituation session with the pixelated habituation video, a mouse was exposed to the pixelated SDS video or pixelated control video.

### Experiment 2: Repeated exposure to SDS videos

After the habituation session, C57BL/6J mice were exposed to a SDS video (stress group; randomly selected from 10 videos) or control (control group) video, then returned to the home cage. This procedure was repeated for 10 consecutive days (Fig. 3a). After each exposure session, all mice were housed individually in standard polyolefin plastic cages. We then examined the effects of repeated video exposure on emotional behaviours. Mice were weighed daily before being exposed to the videos.

### Exposure to SDS video

**Figure 4:**
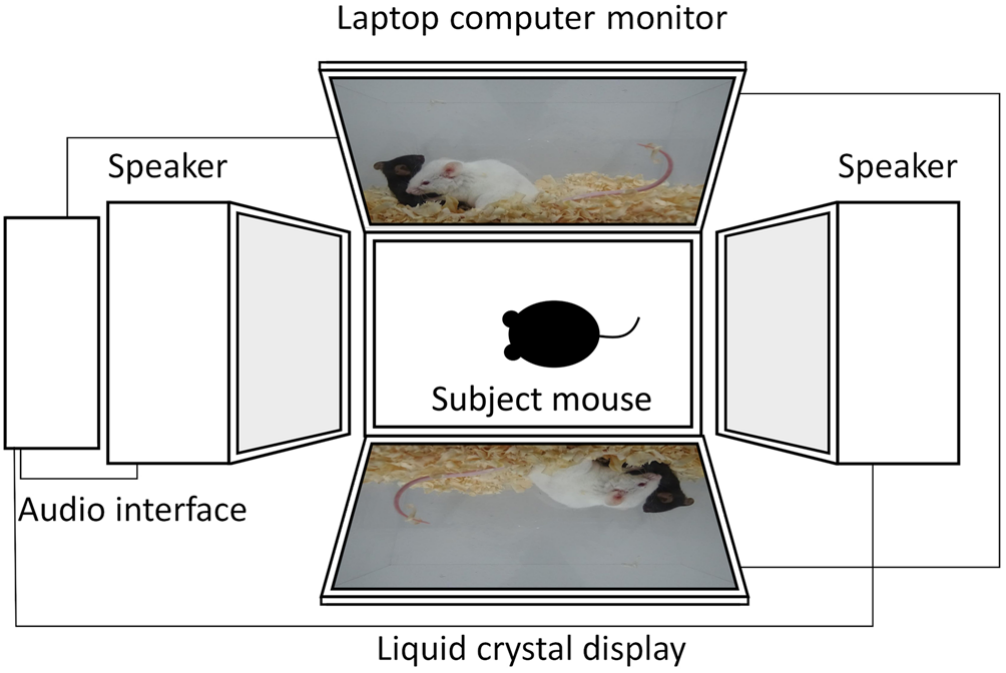
Schematic procedure of exposure to the SDS video. The subject mouse was put into an acrylic cage and exposed to the SDS video from both sides of the cage via a laptop computer monitor and a liquid crystal display.

C57BL/6J mice were individually placed into the acrylic cage, which was sandwiched between a laptop computer LCD monitor and a liquid crystal display (Fig. 4). An audio interface and two speakers were connected to the laptop computer. The speaker volume was adjusted to levels equivalent to those of the actual SDS experiments (about 80 dB at maximum levels).

### Corticosterone assays

The corticosterone assays were performed as previously described^10^. Trunk blood was collected in individual heparin tubes (TERUMO, Tokyo, Japan) 20 min after a single video exposure. Blood samples were centrifuged at 3500 × *g* for 90 s at 4 °C to obtain plasma and stored at − 80 °C until use. Plasma samples were diluted 100 times in assay buffer and analysed using a Corticosterone ELISA Kit (Cayman Chemical, MI, USA) following the manufacturer’s instructions. All samples were measured in duplicate.

### Immunohistochemistry

Immunohistochemical staining was performed as previously described, with minor modifications^37^. Ninety minutes after a single video exposure, mice were anaesthetised with sodium pentobarbital (50 mg/kg) and transcardially perfused with 0.02 M phosphate buffer (PB, pH 7.4) followed by fixative (4% paraformaldehyde in 0.1 M PB) (Fig. 2a). The brains were removed and stored in the same fixative for 24 h at 4 °C, and were subsequently immersed for 2 days at 4 °C in 30% sucrose and 4% paraformaldehyde in 0.1 M PB. Brains were embedded in OCT compound (Sakura Finetek Japan, Tokyo, Japan) and stored at − 80 °C until use. The brains were cut into 40 µm of coronal sections using a microtome (REM-710, Yamato Kohki, Saitama, Japan) and immunohistochemically stained using a free-floating method. The sections were permeabilised with 0.1% Triton X-100 in phosphate-buffered saline (PBST) for 30 min, treated with methanol containing 3% H_2_O_2_ for 5 min, then blocked with PBST containing 3% normal goat serum (PBSTS) for 1 h. Sections were incubated with anti-c-Fos antibody (1:10,000; sc-52, Santa Cruz Biotechnology, TX, USA) in PBSTS for 24 h at 4 °C. After incubation with biotinylated anti-rabbit IgG antibody (1:500; Vector Laboratories, CA, USA) for 1 h at room temperature, sections were incubated with Elite avidin–biotin peroxidase complex (Vector Laboratories) according to the manufacturer’s instructions. The immunostaining reaction was developed using the ImmPACT DAB peroxidase substrate kit (Vector Laboratories). Sections were mounted onto glass slides and air-dried overnight, counterstained with 0.5% methyl green, dehydrated in graded ethanol solutions, cleared with xylene, then coverslipped with VectaMount Mounting Medium (Vector Laboratories). Digital images of brain sections were taken using a microscope (Olympus BX-60, Olympus, Tokyo, Japan) and digitised by a camera (WRAYCAM-NOA2000, WRAYMER, Osaka, Japan). The Allen Brain Atlas^38^ and its mouse brain stereotaxic coordinates were used to define the brain area boundaries. The c-Fos immunopositive nuclei were counted using ImageJ v 1.52a software, developed at the National Institutes of Health^39^. We defined immunopositive nuclei as nuclei that were darker than the threshold determined by adjacent background stains in each brain region, and we counted the immunopositive nuclei within a 200 × 200 μm area.

### Social interaction test (SIT)

The SIT was performed as previously described^26^. First, a C57BL/6J mouse was allowed to explore an open field arena (40 cm × 40 cm) for 2.5 min. Along one side of the arena was a circular wire cage (8 cm diameter) that remained empty during the first trial (target absent). The mouse was then removed from the testing arena and a novel ICR mouse was placed into the wire cage (target present). Last, the C57BL/6J mouse was returned to the arena and allowed to explore. The time spent in the interaction zone (a 14 cm × 25 cm area surrounding the cage) for each session was recorded and analysed using video tracking software (Smart 3.0, Panlab, Barcelona, Spain). The social interaction ratio (SI ratio) was obtained by dividing the time spent in the interaction zone with the target present by the time spent in the interaction zone with the target absent.

### Elevated plus-maze test (EPM)

The EPM was performed as previously described^10^. The maze comprised two perpendicular intersecting runways. One runway had tall walls (closed arms), and the other runway had no walls (open arms). The runways were 5 cm wide and 25 cm long, and the closed walls were 15 cm tall. The maze was 50 cm from the floor. Mice were placed in the central area, facing one of the closed arms, and were allowed to explore for 5 min. The time spent in the open arms was recorded and analysed using video tracking software (Smart 3.0).

### Forced swim test (FST)

The FST was performed as previously described^10^. Mice were placed individually into 5 L beakers (27 cm × 19 cm) containing 3.5 L of water (23 ± 1 °C) for 6 min. An immobile posture was defined as stopping all active behaviours and floating in the water with minimal movement^40^. Movements during the 6 min were recorded using a video camera, and blinded experimenters recorded immobility as when mice spent more time in an immobile posture than in the performance of active behaviours over each 5 s period.

### Sucrose preference test (SPT)

The SPT was performed as previously described^10^. This test was conducted over 3 days, which included a 2-day training period. Mice were trained to drink from two bottles (water and 1% sucrose) for 2 days. The position of the two bottles was balanced across the experimental mice to exclude potential side preference bias. On day 3, mice were deprived of food, water, and sucrose for 4 h. Afterwards, mice were again provided water and sucrose bottles for 1 h, and their liquid consumption was recorded. Sucrose preference was obtained by dividing sucrose consumption by total consumption (sucrose + water).

## Statistics

All data are presented as the mean ± standard error of the mean (SEM). Data were analysed using IBM SPSS Statistics 21 (IBM, NY, USA). For comparing two groups, an unpaired *t*-test was used for normally distributed data, and Mann–Whitney *U* test was used for not normally distributed data. For comparing more than two groups, a two-way repeated-measures analysis of variance (ANOVA) followed by Bonferroni’s *post-hoc* test was used. Differences with *p* < 0.05 were considered statistically significant.

## Data Availability

The datasets generated during and/or analysed during the current study are available from the corresponding author on reasonable request.

## References

[1] Blanchard EB (2004). Studies of the vicarious traumatization of college students by the September 11th attacks: effects of proximity, exposure and connectedness. Behav. Res. Ther..

[2] Cougle JR, Resnick H, Kilpatrick DG (2009). Does prior exposure to interpersonal violence increase risk of PTSD following subsequent exposure?. Behav. Res. Ther..

[3] Holman EA, Garfin DR, Silver RC (2014). Media's role in broadcasting acute stress following the Boston Marathon bombings. Proc. Natl. Acad. Sci. U. S. A..

[4] Van den Berg CL, Lamberts RR, Wolterink G, Wiegant VM, Van Ree JM (1998). Emotional and footshock stimuli induce differential long-lasting behavioural effects in rats; involvement of opioids. Brain Res..

[5] Warren BL (2013). Neurobiological sequelae of witnessing stressful events in adult mice. Biol. Psychiatry.

[6] Sial OK, Warren BL, Alcantara LF, Parise EM, Bolanos-Guzman CA (2016). Vicarious social defeat stress: Bridging the gap between physical and emotional stress. J. Neurosci. Methods.

[7] Cooper SE (2017). Comparison of chronic physical and emotional social defeat stress effects on mesocorticolimbic circuit activation and voluntary consumption of morphine. Sci. Rep..

[8] Finnell JE (2017). Physical versus psychological social stress in male rats reveals distinct cardiovascular, inflammatory and behavioral consequences. PLoS ONE.

[9] Iniguez SD (2018). Vicarious social defeat stress induces depression-related outcomes in female mice. Biol. Psychiatry.

[10] Nakatake Y (2020). The effects of emotional stress are not identical to those of physical stress in mouse model of social defeat stress. Neurosci. Res..

[11] Cruz-Martín A, Huberman AD (2012). Visual cognition: rats compare shapes among the crowd. Curr. Biol..

[12] Chen Q, Panksepp JB, Lahvis GP (2009). Empathy is moderated by genetic background in mice. PLoS ONE.

[13] Jeon D (2010). Observational fear learning involves affective pain system and Cav1.2 Ca2+ channels in ACC. Nat. Neurosci..

[14] Sanders J, Mayford M, Jeste D (2013). Empathic fear responses in mice are triggered by recognition of a shared experience. PLoS ONE.

[15] Langford DJ (2006). Social modulation of pain as evidence for empathy in mice. Science.

[16] Nakashima SF, Ukezono M, Nishida H, Sudo R, Takano Y (2015). Receiving of emotional signal of pain from conspecifics in laboratory rats. R Soc. Open Sci..

[17] Matsuda S (1996). Persistent c-fos expression in the brains of mice with chronic social stress. Neurosci. Res..

[18] Numa C (2019). Social defeat stress-specific increase in c-Fos expression in the extended amygdala in mice: Involvement of dopamine D1 receptor in the medial prefrontal cortex. Sci. Rep..

[19] Nestler EJ, Carlezon WA (2006). The mesolimbic dopamine reward circuit in depression. Biol. Psychiatry.

[20] Yu T (2011). Cognitive and neural correlates of depression-like behaviour in socially defeated mice: an animal model of depression with cognitive dysfunction. Int. J. Neuropsychopharmacol..

[21] Russo SJ, Nestler EJ (2013). The brain reward circuitry in mood disorders. Nat. Rev. Neurosci..

[22] Shackman AJ, Fox AS (2016). Contributions of the central extended amygdala to fear and anxiety. J. Neurosci..

[23] Langford DJ (2010). Coding of facial expressions of pain in the laboratory mouse. Nat. Methods.

[24] de Waal FB (2008). Putting the altruism back into altruism: the evolution of empathy. Annu. Rev. Psychol..

[25] Sakaguchi T, Iwasaki S, Okada M, Okamoto K, Ikegaya Y (2018). Ethanol facilitates socially evoked memory recall in mice by recruiting pain-sensitive anterior cingulate cortical neurons. Nat. Commun..

[26] Golden SA, Covington HE, Berton O, Russo SJ (2011). A standardized protocol for repeated social defeat stress in mice. Nat. Protoc..

[27] De Vry J, Benz U, Schreiber R, Traber J (1993). Shock-induced ultrasonic vocalization in young adult rats: a model for testing putative anti-anxiety drugs. Eur. J. Pharmacol..

[28] Sales GD (1991). The effect of 22 kHz calls and artificial 38 kHz signals on activity in rats. Behav. Processes.

[29] Valenta JG, Rigby MK (1968). Discrimination of the odor of stressed rats. Science.

[30] Kikusui T, Takigami S, Takeuchi Y, Mori Y (2001). Alarm pheromone enhances stress-induced hyperthermia in rats. Physiol. Behav..

[31] Inagaki H (2014). Identification of a pheromone that increases anxiety in rats. Proc. Natl. Acad. Sci. U. S. A..

[32] Willner P, Towell A, Sampson D, Sophokleous S, Muscat R (1987). Reduction of sucrose preference by chronic unpredictable mild stress, and its restoration by a tricyclic antidepressant. Psychopharmacology.

[33] Ayensu WK (1995). Effects of chronic mild stress on serum complement activity, saccharin preference, and corticosterone levels in Flinders lines of rats. Physiol. Behav..

[34] Rush AJ (2011). Combining medications to enhance depression outcomes (CO-MED): acute and long-term outcomes of a single-blind randomized study. Am. J. Psychiatry.

[35] Wadman M (2013). NIH mulls rules for validating key results. Nature.

[36] Warren BL, Mazei-Robison MS, Robison AJ, Iniguez SD (2020). Can i get a witness? Using vicarious defeat stress to study mood-related illnesses in traditionally understudied populations. Biol. Psychiatry.

[37] Yamada M, Saitoh A, Ohashi M, Suzuki S, Oka J (2015). Induction of c-Fos immunoreactivity in the amygdala of mice expressing anxiety-like behavior after local perfusion of veratrine in the prelimbic medial prefrontal cortex. J. Neural Transm (Vienna).

[38] Franklin, K. B. J. & Paxinos, G. The Mouse Brain in Stereotaxic Coordinates (*Academic Press*, 2008).

[39] Schneider CA, Rasband WS, Eliceiri KW (2012). NIH Image to ImageJ: 25 years of image analysis. Nat. Methods.

[40] Porsolt RD, Bertin A, Blavet N, Deniel M, Jalfre M (1979). Immobility induced by forced swimming in rats: effects of agents which modify central catecholamine and serotonin activity. Eur. J. Pharmacol..

